# Cardiomyocyte-Restricted Low Density Lipoprotein Receptor-Related Protein 6 (LRP6) Deletion Leads to Lethal Dilated Cardiomyopathy Partly Through Drp1 Signaling: Erratum

**DOI:** 10.7150/thno.72572

**Published:** 2022-04-26

**Authors:** Zhidan Chen, Yang Li, Ying Wang, Juying Qian, Hong Ma, Xiang Wang, Guoliang Jiang, Ming Liu, Yanpeng An, Leilei Ma, Le Kang, Jianguo Jia, Chunjie Yang, Guoping Zhang, Ying Chen, Wei Gao, Mingqiang Fu, Zheyong Huang, Huiru Tang, Yichun Zhu, Junbo Ge, Hui Gong, Yunzeng Zou

**Affiliations:** 1 Shanghai Institute of Cardiovascular Diseases, Zhongshan Hospital, and Institutes of Biomedical Sciences, Fudan University, Shanghai 200032, China.; 2 State Key Laboratory of Genetic Engineering, Zhongshan Hospital and School of Life Sciences, Fudan University. International Centre for Molecular Phenomics, Collaborative Innovation Center for Genetics and Development, Shanghai 200438, China.; 3 Department of Physiology and Pathophysiology, Shanghai Medical College, Fudan University, Shanghai 200032, China.

In the original publication, errors were regrettably found in Fig. 4A. The mistake was made during the figure arrangement. In Fig. 4A, the original bands of GAPDH were misused twice in two different treatment groups. We have checked the original bands and corrected them. The correction is shown below. The authors declare the amendments do not change the results and conclusions of the paper.

## Figures and Tables

**Figure 4:**
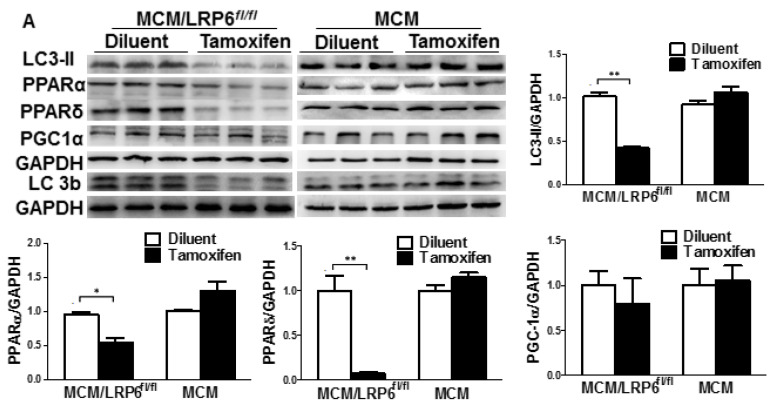
Western blot analysis of LC3-II, PPARα, PPARδ, PGC1α and LC3b (LC3-I: the upper band; LC3-II: the down band) in heart tissue from diluent or tamoxifen-injected MCM or MCM/LRP6^fl/fl^ mice.

